# The Acceptability, Feasibility, and Effectiveness of Wearable Activity Trackers for Increasing Physical Activity in Children and Adolescents: A Systematic Review

**DOI:** 10.3390/ijerph18126211

**Published:** 2021-06-08

**Authors:** Amy V. Creaser, Stacy A. Clemes, Silvia Costa, Jennifer Hall, Nicola D. Ridgers, Sally E. Barber, Daniel D. Bingham

**Affiliations:** 1 School of Sport, Exercise, and Health Sciences, Loughborough University, Loughborough LE11 3TU, UK; s.a.clemes@lboro.ac.uk (S.A.C.); s.costa@lboro.ac.uk (S.C.); 2 Bradford Institute for Health Research, Bradford Teaching Hospitals Foundation Trust, Bradford BD9 6RJ, UK; jennifer.hall@bthft.nhs.uk (J.H.); sally.barber@bthft.nhs.uk (S.E.B.); Daniel.Bingham@bthft.nhs.uk (D.D.B.); 3 Leicester Biomedical Research Centre, National Institute for Health Research (NIHR), University Hospitals of Leicester NHS Trust and University of Leicester, Leicester LE5 4PW, UK; 4 School of Exercise and Nutrition Sciences, Institute for Physical Activity and Nutrition, Deakin University, Geelong 3125, Australia; nicky.ridgers@deakin.edu.au

**Keywords:** physical activity, systematic review, feasibility, interventions, wearable activity trackers, children, adolescents

## Abstract

Wearable activity trackers (wearables) embed numerous behaviour change techniques (BCTs) that have previously been shown to increase adult physical activity (PA). With few children and adolescents achieving PA guidelines, it is crucial to explore ways to increase their PA. This systematic review examined the acceptability, feasibility, and effectiveness of wearables and their potential mechanisms of action for increasing PA in 5 to 19-year-olds. A systematic search of six databases was conducted, including data from the start date of each database to December 2019 (PROSPERO registration: CRD42020164506). Thirty-three studies were included. Most studies (70%) included only adolescents (10 to 19 years). There was some—but largely mixed—evidence that wearables increase steps and moderate-to-vigorous-intensity PA and reduce sedentary behaviour. There were no apparent differences in effectiveness based on the number of BCTs used and between studies using a wearable alone or as part of a multi-component intervention. Qualitative findings suggested wearables increased motivation to be physically active via self-monitoring, goal setting, feedback, and competition. However, children and adolescents reported technical difficulties and a novelty effect when using wearables, which may impact wearables’ long-term use. More rigorous and long-term studies investigating the acceptability, feasibility, and effectiveness of wearables in 5 to 19-year-olds are warranted.

## 1. Introduction

Physical activity (PA) provides physical, psychological, social, and cognitive health benefits throughout a person’s lifespan [1,2,3]. Physical activity during childhood and adolescence is associated with current and future health outcomes such as reduced adiposity [4,5] and enhanced motor skill development [6]. There is evidence that increasing PA can reduce depressive symptoms [7], and higher levels of PA are related to a greater quality of life [8] in children and adolescents. Despite these benefits, physical inactivity is common amongst children and adolescents. An estimated 28% and 57% of adolescent girls and boys (12 to 17-years) in European countries meet the guidelines of 60 minutes of moderate-to-vigorous-intensity PA (MVPA) every day [9].

To date, PA interventions in ≤16 year-olds have produced negligible-to-small increases in total PA and MVPA [10]. Previous evidence suggests that PA interventions that embed numerous behavioural change techniques (BCTs) may be more effective than those that do not [11]. Behavioural change techniques are “observable, replicable, and irreducible component(s) of an intervention designed to alter or redirect causal processes that regulate behaviour” ([12] p. 82). A previous systematic review [11] reported that effective school-based PA interventions aimed at 15 to 19-year-olds had an average of 10.5 (range: 5–20) BCTs compared to ineffective interventions that had an average of 4 (range: 3–6) [11]. Behavioural change techniques unique to effective interventions included self-monitoring, feedback, goal setting, action planning, and social support [11].

Previous research has reported that wearable activity trackers (wearables) and their partnering applications (apps) embed an average of 19 (range: 15–24) BCTs, including self-monitoring, feedback, action planning, goal setting, prompts/cues, and social comparison [13]. Given the number and type of BCTs that wearables embed, wearables may offer an effective way of increasing child and adolescent PA. There is some evidence that wearables are a potentially effective way of increasing PA in adults [14,15,16,17,18,19,20,21]. A meta-analysis found that wearables can significantly increase an adult’s step count, MVPA, and energy expenditure [20]. It was found that multi-component interventions were more effective than those using a wearable alone, and authors have suggested that this may be due to wearables being combined with additional BCTs [20].

However, less is known about the effectiveness of wearables for increasing child and adolescent PA [22]. In 2016, a systematic review [22] identified three intervention studies [23,24,25] investigating the effectiveness of wearables in 5 to 19-year-olds. There was little evidence to suggest that wearables can increase PA in children and adolescents, with Ridgers et al. [22] concluding that further research using rigorous methods was needed. This review aims to update that presented by Ridgers et al. [22], exploring the most recent literature. Given the evidence that PA interventions are most effective the more BCTs they embed [11], this review will identify BCTs in wearable interventions and may provide insights into the potential mechanisms of action behind the level of effectiveness of interventions, which were not explored in the review by Ridgers et al. [22].

The acceptability and feasibility of wearables are also important to consider in order to allow researchers to understand the potential reasons for the level of effectiveness of interventions and how best to implement such interventions [26,27]. For example, similar devices, such as pedometers, have been found to increase PA in 5 to 18-year-olds [28]. However, pedometers require users to manually record their PA (e.g., steps) each day [29,30], which may reduce intervention compliance rates and limit effectiveness [31], whereas wearables provide incremental feedback via a monitoring screen, app, and/or online dashboard, enabling users to easily monitor their long-term PA. This suggests that wearables may be more acceptable and feasible than other similar devices (e.g., pedometers). The review by Ridgers et al. [22] identified two studies [32,33] exploring the feasibility of wearables in 5 to 19-year-olds. There was some evidence that wearables were viewed positively by children and adolescents and that wearables can encourage children and adolescents to be more physically active [22]. However, several barriers to wearable use were identified (e.g., access to technology, lack of comfort) [22]. Although these findings offer some insight into wearables’ acceptability and feasibility, they are based on a small number of studies, and common themes were not identified. This systematic review presents a thematic synthesis to identify key themes related to the acceptability and feasibility of using wearables in children and adolescents. This highlights the most common facilitators and barriers of using wearables and will allow researchers to focus on these factors when developing future wearable-based interventions.

In this review, acceptability refers to constructs outlined by the “Theoretical Framework of Acceptability” [34], including how children and adolescents feel about using a wearable (affective attitude), the amount of perceived effort required to use a wearable (burden), the perceived understanding of how to use a wearable and interpret PA outputs (intervention coherence), and the perceived impact wearables have on children and adolescents’ PA levels (perceived effectiveness). Feasibility refers to the considerations outlined by Abbott [35], such as the facilitators and barriers of using wearables.

This systematic review explores the acceptability, feasibility, effectiveness, and potential mechanisms of action underlying the effectiveness of wearables for increasing PA in children and adolescents (5 to 19 years).

### Research Questions

This systematic review addresses the following research questions:

1. How acceptable are wearables for increasing PA in 5 to 19-year-olds?
2. How feasible are wearables for increasing PA in 5 to 19-year-olds?
3. How effective are wearables for increasing PA in 5 to 19-year-olds?
4. What are the mechanisms of action (BCTs) underlying the influence of wearables on PA in 5 to 19-year-olds?

## 2. Methods

This review is registered with the International Prospective Register for Systematic Reviews (PROSPERO; CRD42020164506) and follows the guidance of the Preferred Reporting Items for Systematic Reviews and Meta-Analyses (PRISMA; [36]).

### 2.1. Search Strategy

A search strategy was developed based on previous reviews [22,37] to identify potentially relevant studies from six databases: PubMed, Web of Science, Scopus, SPORTDiscus, PsycINFO, and ProQuest. Searches took place from the start date of each database to December 2019. Search strategies included the following search strings in four main areas: population (child*, adolescen*, youth, teen*, young person, young people, school child*, family, families, parent*, caregiver, mother, father, home-based, parent–child, parent–adolescent), intervention (intervention, trial, feasibility, pilot, acceptability, program), wearable activity tracker (electronic track*, electronic activ*, electronic activ* monitor*, electronic active* track*, electronic fitness track*, wearable device, wearable act* track*, consumer wearable, wearable, wearable tech*, fitness track*) and outcome (physical act*, energy expenditure, MVPA, steps). The full search strings for each database are presented in Supplementary Materials Table S1. Additional relevant studies were retrieved from the included studies’ bibliographies, which is a method used to identify additional studies in systematic reviews [38].

### 2.2. Inclusion Criteria

Inclusion criteria were developed using the “Population, Intervention, Comparison, Outcomes and Study” approach (PICOS; [36]). The following eligibility criteria were used: (a) published in English; (b) included participants aged 5 to 19-years; (c) examined the use of a wearable within an intervention, acceptability, or feasibility study; (d) measured PA (effectiveness) and/or experiences of using a wearable (acceptability/feasibility); and (e) were a full-text paper, using primary data, published in a peer-reviewed academic journal. All populations (e.g., clinical, overweight/obese), study designs, and settings were included. For acceptability/feasibility studies, experiences of using a wearable could be reported by children and adolescents (5 to 19-year-olds) or others (e.g., adults, such as parents) on behalf of the study population.

For the purpose of this review, wearables were defined as a commercially available wearable device, with the capability of tracking PA (e.g., accelerometry, steps) and at least one other feature (e.g., distance travelled, gamification/rewards) or multiple dimensions of PA (e.g., steps and PA intensities). The wearable needed to provide momentary tracking to gather incremental feedback via a monitoring display, online dashboard, or partnering application, beyond a traditional step-only display [22,39]. Wearables could be used in conjunction with a partnering application/online dashboard or alone, given that smartphone ownership is minimal in younger children [40].

### 2.3. Exclusion Criteria

Studies were excluded if (a) participants were <5 years and >19 years of age and (b) the wearable was not utilised as an intervention/feasibility tool (e.g., measurement tool).

### 2.4. Study Selection and Data Extraction

Study eligibility was independently assessed through title, abstract, and full text-screening by two reviewers (A.V.C. and D.D.B.) using a standardised, unblinded approach. Studies were screened independently, and any disagreements were resolved at the end of each stage by A.V.C. and D.D.B., with any persistent disagreements resolved by a third reviewer (S.C.). If insufficient information was provided, the author(s) of the identified studies were contacted by email to provide relevant information for eligibility assessment.

The first reviewer (A.V.C.) used a standardised form to extract data using an adapted version of the Cochrane data collection form for intervention reviews: RCTs and non-RCTs [41]. The following data were extracted: country of study, sample demographics (e.g., age, sex, ethnicity, weight status, socioeconomic status), study characteristics (e.g., design, description, no. of study arms, length, follow-up period(s), setting), measurement characteristics (e.g., measurement tool, reported outcome), device characteristics (e.g., device brand and model), and study results.

### 2.5. Behaviour Change Techniques (BCTs)

Two reviewers (A.V.C. and J.H.), used the Behaviour Change Technique Taxonomy v1 (BCTTv1), a 93-item coding framework [12], to independently code BCTs present in included studies that measured PA as an outcome of using a wearable (effectiveness studies). Behaviour change techniques present in the wearable, partnering app/online dashboard, and additional study/intervention components were coded. Control and comparator groups of effectiveness studies were also coded for BCTs. Each BCT was coded as present beyond all reasonable doubt (++), present in all probability (+), or absent (−), as recommended by Michie et al. ([12,42], https://www.bct-taxonomy.com/; accessed on 1 June 2020). Techniques were coded as present beyond all reasonable doubt (++) if the study authors provided detailed evidence that the BCT was applied to the target behaviour and population and explained how the BCT was used to promote PA. Techniques were coded as present in all probability (+) if there was mention of the BCT being used without detailed explanation of how the BCT was used, there was some evidence that the BCT was utilised without explicit mention of the BCT by the study authors, or if the wearable(s) had a BCT present, as stated in the study or by manufacturers, using official websites and device manuals, but it was unclear from the study whether they were utilised. The frequency and duration of BCT use/delivery was not coded.

### 2.6. Risk of Bias

The included studies that measured PA as an outcome of using a wearable (effectiveness studies) were assessed for Risk of Bias (RoB) by two reviewers (A.V.C. and D.D.B.) independently. The risk of bias criteria were utilised in a previous systematic review [22], which was adapted from the Methods Guide for Comparative Effectiveness Reviews [43]. The following eight criteria were used: (1) participants were randomly allocated, (2) minimal missing data, (3) data were analysed according to group allocation, (4) the study population was representative of the population of interest, (5) the timing of outcome assessments was similar in all groups, (6) the study reported the validity of the wearable, (7) the study reported the reliability of the wearable, and (8) the study was conducted independently of the wearable manufacturer. Each criterion was scored as yes (1) or no (0). If unclear, the criterion was scored as no (0). Table S2 provides further descriptions of each criterion. An overall score out of 8 was provided, and effectiveness studies were categorized as having a high (score 0–2), medium (score 3–5), and low (score 6–8) risk of bias (adapted from Lewis et al. [18]).

### 2.7. Data Synthesis

To synthesise the data, studies were split into “effectiveness studies” and “feasibility/acceptability studies”. Effectiveness studies were those that provided an outcome measure of PA. The term “effectiveness studies” was chosen as some studies that measured PA did not claim to be an intervention. Studies that reported the experiences of children/adolescents using the wearable, but did not measure PA, were considered feasibility/acceptability studies. A narrative review was conducted for effectiveness studies, given that the heterogeneity of study designs and outcomes did not permit a meta-analysis. A thematic synthesis was conducted for feasibility/acceptability studies in which the authors provided quotations from participants and/or the authors’ interpretations of participant experiences using a wearable (as used previously by Fletcher et al. [44]). The thematic synthesis followed the three stages outlined by Thomas and Harden, which are presented in Table 1 [45].

Line-by-line coding was carried out by one reviewer (A.V.C.) using the NVivo software (QSR International, Melbourne, Australia), and the meaning and content of each code was agreed upon by a second reviewer (J.H.). One reviewer (A.V.C.) developed descriptive themes from the codes by grouping those that were similar together and further developed these into analytical themes by relating them to the reviews’ aims. These codes were discussed and refined with the help of J.H. This approach has been used previously [44] and was chosen as an inductive approach was taken.

## 3. Results

### 3.1. Search Results

**Figure 1:**
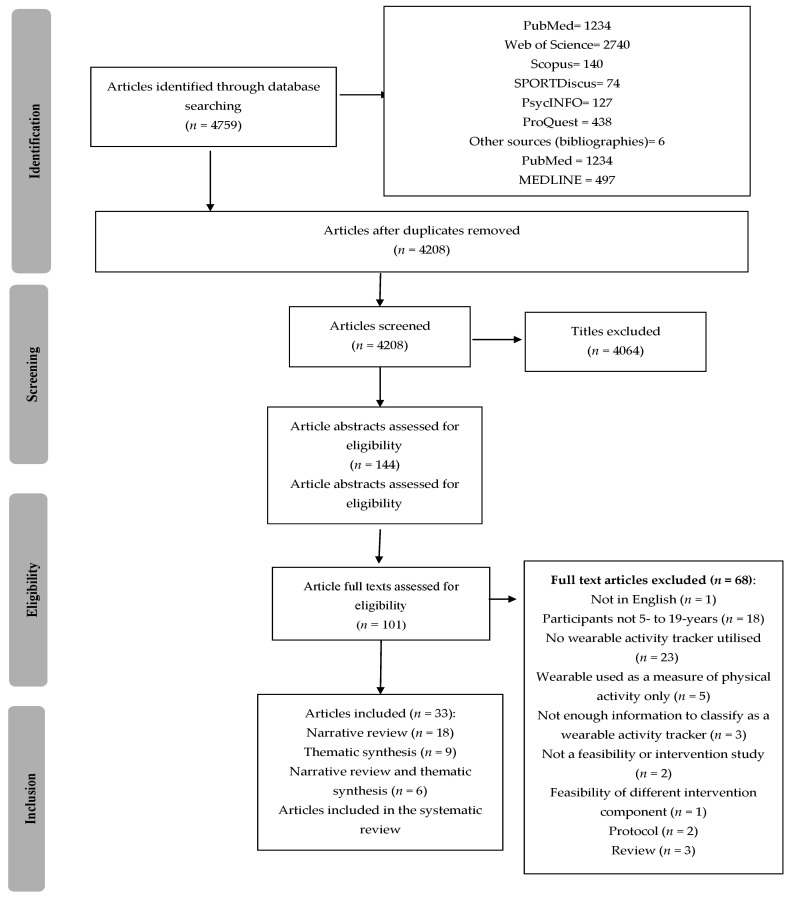
Preferred Reporting Items for Systematic Reviews and Meta-Analyses (PRISMA) flow diagram illustrating the review process.

Figure 1 outlines the PRISMA diagram. A total of 4759 studies were retrieved (4208 after removing duplicates). Thirty-three studies were eligible for inclusion. Eighteen were included in the narrative review (investigating effectiveness), nine were included in the thematic synthesis (investigating acceptability/feasibility), and six were included in both the narrative review and thematic synthesis (investigating acceptability/feasibility, and effectiveness). Table 2 presents descriptions of the included studies.

### 3.2. Countries

Most studies were conducted in the USA (*n* = 16; 48.5%). The remaining studies were conducted in England (*n* = 4; 12.1%), Australia (*n* = 3; 9.1%), Canada (*n* = 2; 6.1%), Germany, Finland, Poland, Netherlands, and New Caledonia (all *n* = 1; 3.0%). Three studies (9.1%) did not specify the country.

### 3.3. Population

Studies involved 1843 participants (range: 6–196). Most studies targeted only adolescents (10 to 19 years) (*n* = 23, 70%), with eight (24%) focusing on children and adolescents (5 to 19 years) and two (6%) on only younger children (5 to 9 years). Seventeen studies (51.5%) recruited participants from specific demographic groups (e.g., overweight/obesity, low PA, ADHD, cancer survivors, rural communities).

### 3.4. Study Design

Study periods ranged from 4 days [62] to 6 months [58,72]. Most studies were pilot or feasibility studies (*n* = 25; 75.8%), four (12.1%) were quasi-experimental, and four (12.1%) were RCTs. Of the 24 effectiveness studies, most had one study group (*n* = 18; 75%), with six (25%) comparing two study groups, of which one group did not use a wearable [69]. Sixteen effectiveness study groups used a wearable as part of a multi-component intervention (55.2%), and 13 (44.8%) used a wearable on its own. The most common additional component was technology-based (*n* = 5; 41.7%), with three studies using a social media platform [63,69,70].

### 3.5. Devices

Most studies used one wearable (*n* = 30; 90.9%). Twenty-one device models were used, with Fitbit devices being the most used brand (*n* = 17; 51.5%). Fifteen devices were wrist-worn (71.4%), five devices were attached to clothing (23.8%), and one device was worn on the upper arm (4.76%). One study [66] did not report the brand or model of the device.

### 3.6. Risk of Bias

**Figure 2:**
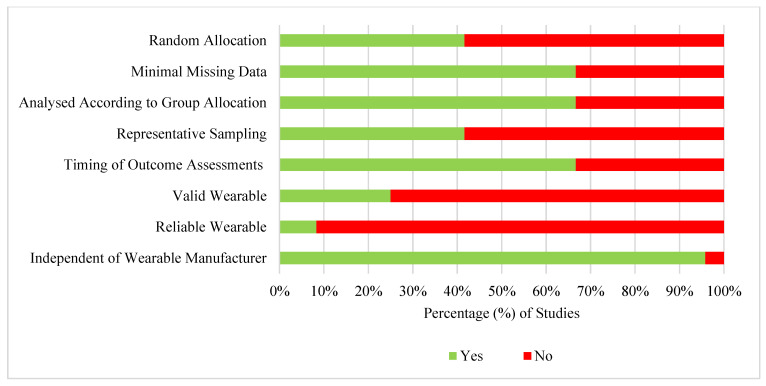
Summary of the risk of bias results across all criteria for effectiveness studies (*n* = 24).

Figure 2 and Table 3 provide a summary of the RoB results for the effectiveness studies. Six (25%), thirteen (54.2%), and five (20.8%) effectiveness studies were deemed to show a low, medium and high RoB. The most common high-risk practices were not reporting the validity (75%) and reliability (92%) of the wearable. The most common low-risk practice was a study being conducted independently of the wearable manufacturer (96%).

### 3.7. Behaviour Change Techniques (BCTs)

**Figure 3:**
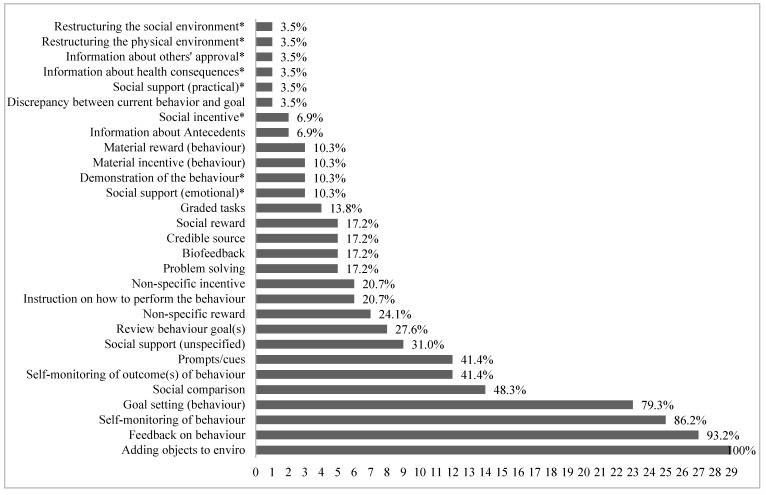
Total number of wearable groups with BCTs present in studies investigating effectiveness (*n* = 29). * Present in multi-component study groups exclusively.

Figure 3 displays the number of BCTs present in the effectiveness studies. Behavioural change techniques were coded for 29 groups (groups using a wearable). Wearable studies investigating effectiveness had a mean of 7.8 (range: 2–12) BCTs. Sixty-four (68.8%) BCTs were not used in any effectiveness studies. Multi-component groups had on average 9.6 BCTs, compared to an average of 6.3 BCTs for those using a wearable alone. Eight BCTs were unique to multi-component groups. Four control groups (30.8%) had one BCT present [54,63,65]. One study had a comparator intervention group (no wearable), with eight BCTs present [69].

### 3.8. Outcomes

Of the 24 effectiveness studies, 14 (58.3%) used a wearable as an outcome measure, three (12.5%) used self-reporting, two (6.1%) used a research-grade monitoring-device (e.g., accelerometer), two (8.3%) used both a wearable and self-report, and three studies (12.5%) used a wearable and a research-grade monitoring-device. Most studies (*n* = 17; 70.8%) reported more than one PA outcome. Table S3 displays how the studies measured and defined PA outcomes.

### 3.9. Effectiveness of Wearables on Physical Activity Outcomes

Of the 24 studies examining the effectiveness of wearables for increasing PA in children and adolescents, a range of different outcome measures were used. The findings of these studies, according to the different outcomes, are summarised below. Findings are stratified by the number of BCTs included in the intervention (above or below the mean number of BCTs, which was eight in this review (see Section 3.7), and, where applicable, age group (children versus adolescents).

#### 3.9.1. Step Count

Fifteen studies measured step count [23,25,47,48,50,51,53,58,59,64,66,67,70,72]. Most studies had a medium RoB (*n* = 10; 66.7%). Participants recorded an average of 8166 daily steps throughout the study periods [25,48,50,51,64,66,70,72].

Step count: ≥8 BCTs: Eight studies measuring step count incorporated ≥8 BCTs [23,25,51,58,59,64,66,70]. Two studies had child participants (5 to 9 years), with one finding that children took 630 more steps when using a wearable than in a typical recess period (not tested statistically) [23]. No change in step count was found in the second study [64]. Three studies had adolescent participants (10 to 19 years) [51,66,70]. One study found a significant increase in steps (+ 107 steps/day) [70] and one study found that the least active participants increased their daily steps by 15% [51] during intervention periods. There were no differences in step count between adolescents using a wearable on their own and adolescents using a wearable alongside their parent [66]. Three studies had both child and adolescent participants (5 to 19 years) [25,58,59]. One study found those participating in the intervention took significantly more steps (+ 2500 steps) than controls [59], whereas the two remaining studies found no significant change in step count [25] nor any differences in step count between the intervention and control group [58].

Step count: <8 BCTs: Five studies measuring step count incorporated <8 BCTs [47,50,53,67,72]. Four studies had only adolescent participants (10 to 19 years) [47,50,67,72], and the remaining studies had both child and adolescent participants (5 to 19 years) [53]. Two studies found no difference in step count from pre- to post-intervention [47] or between the intervention and control group [53]. One study found that step counts increased by 381 steps/day from intervention week 1 to week 4, but this was not tested statistically [50]. Adolescents participating in a multi-component intervention (wearable and acceptance-based behaviour counselling, with six BCTs), had a significantly higher step count throughout the intervention, which was not found in those receiving a wearable alone (with three BCTs) [67]. One study found a decrease in step count from pre- to post-intervention (−1361 steps/day, not tested statistically) [72].

Step count: ≥8 BCTs and <8 BCTs: Two studies measuring step count compared the results of adolescents participating in one of two intervention groups: one with ≥8 BCTs and one with <8 BCTs [48,50]. One study found higher step counts (not tested statistically) for adolescents receiving a multi-component intervention (wearable, step challenge, and video-based PA exercise sessions (with 11 BCTs)) than those receiving a wearable alone (with seven BCTs) [48], whereas the other study found no difference between adolescents receiving only a wearable (with four BCTs) and adolescents receiving a wearable alongside goal-setting and a behaviour change session (with 10 BCTs) [50].

#### 3.9.2. Achievement of Step Goals

Four studies measured the achievement of daily step goals [46,51,53,72]. Most studies had a medium RoB (*n* = 3; 75%) and had adolescent participants [46,51,72] or both children and adolescent participants [53]. Three studies had <8 BCTs (5–7 [46,53,72]) and one study had 8 BCTs [51]. Daily step goals were personalised [46,53] at 10,000 [46,72], 11,000 [51] or 12,000 steps/day [46]. Participants achieved step goals on 35–54% of intervention days [46,51]. There were no differences in the achievement of step goals between the intervention and control groups [53]. One study reported that two participants (8.3%) achieved the step goal of 10,000 steps/day for at least half of the intervention days in months 1 and 2 [72].

#### 3.9.3. Moderate-to-Vigorous Physical Activity (MVPA)

Fourteen studies reported MVPA [23,24,47,50,52,54,55,56,59,63,64,66,67,69]. Most studies measured MVPA in mins/day (*n* = 10, 71.4%), with the remaining reporting the number of days which included MVPA [47], the number of 30-minute bouts of MVPA per day [55], and the time spent in MVPA during recess [23] and school hours [59]. Six studies (42.9%) had a medium RoB, five studies (35.7%) had a low RoB, and three studies (21.4%) had a high RoB. Ten studies measured MVPA from pre- to post-intervention [24,47,50,52,55,56,59,63,67,69].

MVPA: ≥8 BCTs: Nine studies measuring MVPA incorporated ≥8 BCTs [23,24,54,56,59,63,64,66,69]. Two studies had child participants (5 to 9 years) [23,64]. Children achieved a mean of 83 ± 18 mins of daily MVPA whilst using the Garmin vivofit jr [64]. In six 8-year-olds, minutes spent in MVPA during recess increased from the control to intervention period by 21% (not tested statistically) [23]. Six studies had adolescent participants (10 to 19 years) [24,54,56,63,66,69], and one study had both child and adolescent participants [59]. Three studies found no differences in time spent in MVPA between the intervention and control group [24,63,69]. Two studies found participants in the intervention group spent significantly more time in MVP (+ 4.99–6.14 mins/day [54] and + 5 mins/day) [59] than the control group. One study found adolescents in the intervention group participated in an average of 15.26 mins/day of MVPA, which was significantly greater than those participating in the active control (9.12 mins/day) and passive control (10.27 mins/day) groups [54]. One study found no significant differences in time spent in MVPA throughout an intervention [66], whereas one study found time spent in MVPA decreased significantly by 9.5 mins/day [56].

MVPA: < 8 BCTs: Four studies measuring MVPA incorporated <8 BCTs [47,52,55,67]. All four studies had adolescent participants. In two studies, there was no change in the number of days spent participating in ≥60 mins of MVPA from pre- to post-intervention [47] or the number of bouts of MVPA throughout the intervention period [55]. One intervention found a significant average daily increase of 15 mins/day from pre- to post-intervention [52], and one study found that those receiving a wearable alone significantly increased their time spent in MVPA by 11.99 mins/day (with three BCTs), and those receiving multi-component intervention significantly increased their time spent in MVPA by 7.25 mins (with six BCTs) [67].

MVPA: ≥8 BCTs and <8 BCTs: One study measuring MVPA compared the results of adolescents participating in one of two intervention groups: one with ≥8 BCTs and one with <8 BCTs [50]. There were no significant differences in the minutes of MVPA between those receiving a wearable alone (with four BCTs) and those receiving a wearable alongside goal-setting and a behaviour change session (with 10 BCTs) [50].

#### 3.9.4. Light, Moderate, and Vigorous-Intensity Physical Activity (LPA, MPA, VPA), Sedentary Time, and Metabolic Equivalents (METs)

Seven studies reported LPA, MPA, VPA, sedentary time, or total METs [24,48,51,55,58,63,69]. Most studies had a medium RoB (*n* = 5; 71.4%) and incorporated ≥8 BCTs (*n* = 5; 71.4%) [24,51,58,63,69]. One study found that adolescents participated in 64, 14 and 7 mins/day of LPA, MPA, and VPA, respectively [51]. Four (57.1%) studies reported at least one favourable effect, most commonly a reduction in sedentary behaviour [24,48,58,69]. Three studies found that time spent sedentary was significantly lower in the adolescents participating in the intervention group or a subsample (adolescent boys [24]) than the control group [24,58,69], whereas one found no difference [63]. Time spent sedentary was significantly lower for participants receiving Facebook-delivered lifestyle counselling and a wearable (with 11 BCTs) (but not those receiving Facebook-delivered lifestyle counselling alone, with eight BCTs) than the control group during weekdays [69] and for participants receiving a wearable, a step challenge, and video-based PA exercise sessions (with 11 BCTs) than those receiving a wearable alone (with seven BCTs) [48]. Of the three studies measuring pre- and post-intervention LPA, MPA, VPA, and/or METs, one reported a significant increase in MPA for adolescent girls receiving a wearable from baseline to 3 months post-intervention (but not at an 8 month follow-up) compared to the control group [24]. The remaining two studies [55,69] did not report any changes in pre- to post-intervention LPA, MPA, VPA, and/or METs.

#### 3.9.5. Total Physical Activity

One medium-RoB study reported that self-reported PA did not significantly differ between 9 to 12-year-olds in the intervention and control groups (with nine BCTs) [58].

#### 3.9.6. Active Minutes

Four medium-RoB studies reported active minutes [46,48,53,58]. Studies had adolescent participants [46,48] or both child and adolescent participants [53,58]. There were no differences in total active minutes [53], the achievement of active minute goals [53], and easy minutes [58] between intervention and control groups. Adolescents achieved their daily active goal on 55% of intervention days, achieving a mean of 101 active daily minutes by the last intervention week [46]. Adolescents given a wearable, step challenge, and video-based PA exercise sessions (with 11 BCTs) spent more daily minutes being fairly active and very active (not tested statistically) than those receiving a wearable alone (with seven BCTs) [48].

#### 3.9.7. Calorie Expenditure

Two low-RoB studies reported the calories expended in adolescents [65,66]. Calories expended did not differ between intervention groups (wearable alone and wearable with parent, with 11 BCTs for both groups [66]) or the control group [65]. There was some evidence that adolescents who continued using the wearable burned more calories than those that did not [66].

### 3.10. Thematic Synthesis

Fifteen studies [32,33,49,50,57,58,60,61,62,63,65,67,68,71,72] were included in the thematic synthesis. Eleven [33,49,57,58,60,61,62,63,65,68,71] provided quotations from participants. Table 4 provides an overview of the themes identified, along with findings and supporting quotations. The inductive line-by-line coding resulted in 182 codes, which were developed into four analytical themes embedding 13 subthemes.

## 4. Discussion

This systematic review investigated the acceptability, feasibility, effectiveness, and potential mechanisms of action underlying the effectiveness of wearables for increasing PA in children and adolescents (5 to 19 years). Thirty-three studies were identified, with 18 investigating effectiveness, nine investigating acceptability/feasibility, and six investigating both the acceptability/feasibility and effectiveness of wearables.

### 4.1. Effectiveness of Wearables on Physical Activity Outcomes

This review found that half of all effectiveness studies reported some evidence that wearables may increase PA outcomes (steps, MVPA) and reduce sedentary time. However, the evidence was mixed. There were no apparent differences in wearables’ effectiveness on steps or MVPA between interventions incorporating ≥8 BCTs, and <8 BCTs. There was mixed evidence that multi-component interventions that had more BCTs were more effective at increasing step counts and MVPA than using wearables on their own. However, it was found that multi-component interventions may be more effective at reducing sedentary behaviour than using wearables on their own. The heterogeneity of the study samples, design, and PA outcomes did not permit a meta-analysis to be conducted. Thus, it is difficult to determine what intervention approach may be effective and why the evidence was mixed. In contrast to this review’s findings, a meta-analysis exploring the effectiveness of wearables on adult PA and sedentary behaviour [20] found multi-component interventions had a greater effect on PA than those using wearables alone [20], and there was no evidence that wearables reduced sedentary time in adults [20]. Potential reasons for the differences between the findings of this review and previous reviews in adults may be due to the types of BCTs used and children/adolescents’ ability to understand and utilise the included BCTs, which is discussed in Section 4.2. However, there is a need for more controlled trials to establish whether multi-component interventions produce different PA outcomes in children and adolescents and adults than using wearables alone [20].

Despite this review’s aim of exploring wearable effects on child (5 to 9 years) and adolescent (10 to 19 years) PA, most studies included only adolescent participants (*n* = 23, 70%), with two studies (6%) including only child participants. Thus, caution should be taken when generalising results to all age groups (5 to 19-year-olds). A previous systematic review found that smartphone-based interventions were more effective at increasing child PA than adolescent PA [73]. It was suggested that younger participants would have required parental assistance to use the technology, which may have encouraged parental involvement and the monitoring of their child’s PA behaviours [73]. One study included in this review found no difference in daily steps, MVPA, or calorie expenditure between adolescents provided with a wearable alone and adolescents and their parent receiving a wearable [66]. Therefore, the potential addition of parental involvement in child-based wearable interventions may result in different PA outcomes to adolescent-based wearable interventions, in which parental involvement may have less of an influence. However, it is unclear whether there would be any differences in wearables’ effectiveness based on age groups as the included studies with child and adolescent participants did not stratify their results based on age groups.

Despite the mixed evidence regarding the effectiveness of wearables, the thematic synthesis revealed that some participants perceived an increase in their PA levels. This perceived increase in PA has been found in adult wearable users [39,74,75]. However, participants from three studies suggested that a wearable did not increase their PA, as they were already active. One study found adolescents in the adoption stage (participating in regular PA) of behaviour change increased their MVPA, whereas those in the preadoption stage (not participating in regular PA, with no/little intention to do so) did not [52]. This evidence suggests that wearables may be more effective for individuals who are already active. There is some evidence that PA interventions in general attract typically active individuals [76,77], thus reflecting a greater problem regarding PA promotion. In this review, four studies recruited inactive participants [24,49,67] or those with low motivation to be physically active [65]. There was some evidence that wearables increased inactive children/adolescents’ PA (MVPA [67], and MPA for girls [24]); thus, future research could benefit from engaging less active children/adolescents in wearable interventions.

### 4.2. Mechanisms of Action

On average, 7.8 BCTs were present in studies investigating the effectiveness of wearables. The most included BCTs (other than adding an object to the environment (wearable)) were feedback on behaviour, self-monitoring of behaviour, and goal setting. Previous evidence suggests that interventions incorporating feedback, self-monitoring, and goal setting are more effective at increasing PA than those without these components [11,78], prompting both short (≤6 months) and long-term (≥12 months) behaviour change [79]. The thematic synthesis found that children and adolescents perceived feedback, self-monitoring, and goal setting as an acceptable way of increasing their PA. However, with regards to effectiveness, there were no apparent associations between the type of BCTs and study effectiveness, given that most studies utilised these BCTs and mixed evidence was found. Discrepancies may be due to users’ ability to utilise the BCTs and the frequency and duration of the used BCTs. This is reflected in the thematic synthesis, whereby some participants suggested a lack of understanding of how to interpret PA data and use device features, such as setting goals [49]. Future interventions may benefit from supporting the use of BCTs and investigating the frequency and duration for which participants use BCTs.

Competition was identified as a potential mechanism of action underlying wearables’ impact on PA. Participants reported competition with friends and trying to beat their previous PA scores/goals. Previous research has found that adults also use competition as a way of increasing PA when using a wearable [39]. An interesting finding from the thematic synthesis was that competition, alongside goal setting, may discourage PA, creating feelings of pressure and guilt [57]. In one study, adolescents perceived that autonomy, relatedness, and competence decreased after using the Fitbit Charge [57]. Qualitative findings suggested that competition and predetermined goals may have contributed to these findings [57]. According to the self-determination theory, for a behaviour (e.g., PA) to occur, an individual must be motivated to perform the behaviour [57,80]. This motivation is supported by a person’s autonomy, relatedness, and competence [80]. Thus, the reduction in these three constructs [57] may suggest that wearables have a negative effect on motivation to be physically active. However, an online survey found that wearables resulted in minimal negative psychological consequences in current and previous adult wearable users [81]. More research is needed to explore the impact of wearables on child and adolescent mental health and well-being and to investigate how wearables may be best used to minimise the negative impact on an individual’s perceived autonomy, relatedness, and competence to be physically active.

A recent study found that wearables and their partnering apps had an average of 19 out of 93 BCTs (range: 15–24) [13]. This compares to the total of 7.8 found in the current review. Thus, the included studies did not utilise all BCTs that are already embedded in wearables. Indeed, 64 (68.8%) of the 93 BCTs were not used in any included effectiveness study, despite previous research confirming that some BCTs (e.g., action planning) are embedded in most wearables [13]. Some absent BCTs, such as action planning and self-talk, have previously been found to increase child and adolescent [11,82] and adults’ PA [83]. Future research may benefit from combining additional BCTs into wearable interventions, in addition to utilising the BCTs already embedded in wearables. A meta-analysis demonstrated the effectiveness of combining certain BCTs [84]. Interventions were less effective when providing feedback without instructions on how to perform the behaviour and most effective when combining BCTs that corresponded to providing information about health consequences, action planning, and prompts/cues [84].

### 4.3. Acceptability and Feasibility

The acceptability and feasibility of wearables are important for the engagement and effectiveness of an intervention [85]. This review found some evidence that wearables are acceptable and feasible for increasing PA in children and adolescents. The Technology Acceptance Model (TAM [86]) is a commonly used model for understanding users’ acceptance of technology [86,87,88]. The TAM suggests that perceived ease of use and perceived usefulness predict the intention to use technology and in turn actual technology use [49,88]. Perceived ease of use and usefulness of using wearables significantly predicted wearable use in adult users [89].

#### 4.3.1. Perceived Ease of Use

Perceived ease of use is defined as the degree to which a person believes that using a system (wearable) would be free of effort [88]. There is some evidence that wearables were easy to use. One parent reported only using PA outputs that were easy for them to understand, such as steps and distance travelled, as they reflected everyday terminology compared to device-specific terminology (e.g., sqoins; [62]) [60]. Adults have suggested steps to be the most useful type of PA information provided by wearables [74,75]. However, some wearable features (e.g., partnering app challenges, goal setting [49,62]) were deemed difficult to use. The cost of using a wearable (e.g., mobile data allowance) was considered a barrier, regardless of an individual’s socio-economic status [52,68]. When access to technology was available, charging and syncing devices were considered burdensome, and some participants stopped carrying out these tasks. Researchers may have to support individuals to overcome these barriers, such as syncing and charging devices, or prompting participants via action planning techniques/reminders. However, this may reduce the feasibility of large-scale studies by increasing the burden on researchers.

#### 4.3.2. Perceived Usefulness

Wearables were perceived as a useful (the degree to which users believe the wearable would enhance their performance; e.g., PA [88]) way to increase some, but not all, users’ PA (Section 4.1). The reported novelty effect may reflect a reduction in wearables’ perceived usefulness to increase PA in children and adolescents. This reported novelty effect corresponds to device adherence rates declining after 2–4 weeks of use [52,57,68]. It is unclear why this novelty period exists; however, some adults have reported stopping using a wearable due to boredom [90] or feeling that they learned everything they needed from the initial use of the wearable [74]. However, in one study included in this review, adolescents reported not having a safe space to be active [33]. This reflects general barriers to PA beyond wearable use and may contribute to the novelty effect. To encourage long-term device use, the wearable must support users’ pre-existing personal and social contexts [39]. Researchers should not only consider how to support wearables’ perceived usefulness but how to overcome individual barriers to PA.

### 4.4. Strengths and Limitations

A strength of this systematic review was the ability to update a previous review [22], given the progression in research. Despite this, the quality of these studies does not appear to have progressed, and due to the heterogeneity of study designs and the measuring and reporting of PA outcomes, a meta-analysis was unable to be carried out. Most studies were pilot/feasibility studies (*n* = 15), had short durations (with a mode of 4 weeks), lacked control groups (*n* = 12, 50%), and varied in terms of how PA was measured (21 different measurement tools). Physical activity outcomes were measured and defined differently (e.g., studies defined MVPA in nine different ways), and it was unclear how some studies defined outcomes (MVPA [64,66], MPA, VPA, easy minutes [58], and active minutes [46,53,58]). Nineteen (79.2%) studies used a wearable as a measure of PA, as well as the intervention/feasibility tool. Given the most common high-risk practices were the lack of reporting of the validity (75%) and reliability (92%) of wearables and that wearables overestimate step counts in children and adolescents [91,92], there is a need for more rigorous studies to evaluate the long-term effectiveness of wearables for children and adolescents’ PA.

Most studies (*n* = 23, 70%) had only adolescent participants (10 to 19 years); thus, the generalisability of the results to younger age groups (5 to 9 years) is limited. Future research would benefit from investigating wearable acceptability, feasibility, and effectiveness in 5 to 9-year-olds, and where applicable, stratify results based on age groups.

A strength of the current review was the mixed-method approach utilised. Mixed-method studies are widely used to develop PA interventions [93,94,95] and can help in understanding the mechanisms of action behind intervention effects [96]. In particular, integrating the quantitative and qualitative findings (the 1 + 1 = 3 approach [97]) rather than interpreting them separately is recognised as a way of enhancing the value of mixed-methods research [98]. Additionally, BCT coding had the potential to identify “active ingredients” that may create more effective future PA interventions [42]. The BCTTv1 offers an extensive list of well-defined and distinct BCTs [12], which can be mapped on to components of the Behaviour Change Wheel (e.g., Theoretical Domains Framework (TDF) and COM-B model), enabling a systematic way of developing future PA interventions and policies [42]. Unfortunately, the current review found no apparent differences between the number of BCTs utilised and the effectiveness of interventions. It may be that differences in BCT engagement (the frequency and duration of BCTs) rather than the presence (versus absence) of BCTs influenced the effectiveness of interventions. However, the included studies did not report the frequency and duration of BCTs, limiting the ability to code BCT engagement in the current review.

### 4.5. Conclusions

This review observed that approximately half of all effectiveness studies found some evidence that wearables can increase steps and MVPA and reduce sedentary behaviour; however, most evidence was mixed. There were no apparent differences in the effectiveness of interventions based on the number of BCTs used and between studies using a wearable alone or as part of a multi-component intervention. There was some evidence that wearables are acceptable for some children and adolescents, but technical difficulties, device designs, and the novelty effect of using these devices may impact their use. There were mixed perspectives on whether wearables increased PA. Most studies (70%) included only adolescent participants; therefore, caution should be taken when generalising results to younger children. More rigorous (e.g., RCTs and valid and reliable measures of PA) and long-term studies are required. Furthermore, future research would benefit from reporting the duration and frequency in which BCTs are used and investigating the acceptability, feasibility, and effectiveness of wearables in younger age groups (e.g., 5 to 9-year-olds). Further research should consider how to utilise wearable features that motivate individuals, encourage long-term use, limit negative feelings, and overcome barriers to using wearables, as well as barriers to general PA participation.

## Figures and Tables

**Table 1:** Stages of thematic synthesis [45].

Stages of Thematic Synthesis	Description
Line-by-line coding of the findings of primary studies.	Code each line of text according to its meaning and content.
2. The development of “descriptive” themes from the free codes.	Consider the similarities and differences between free codes (stage 1) and group them together to develop descriptive themes.
3. The development of “analytical” themes from the descriptive themes.	Develop themes that “go beyond” the descriptive themes, by addressing how the themes relate to the review aim and generate additional understanding of concepts and hypotheses.

**Table 2:** Summary of included studies.

Citation	Location	Participants	Wearable Model	Review Question Addressed	Study Design	Study Description	Study Duration	BCTs *(n)*	Key PA Findings
Bianchi-Hayes et al. [46]	NY, USA	Nine parent–adolescent (14–16 years, BMI^a^ ≥85 percentile) dyads.	Jawbone UP MOVE	Effectiveness	One-arm pilot study.	Adolescents and their parents received a Jawbone UP MOVE. Participants worked with researchers to identify new activities.	10 weeks	5	Participants achieved their step and active minute goals on 35–39% and 55% of intervention days, respectively.
Bronikowski et al. [47]	Poznań, Poland	196 participants (M = 11.5–17.2 years) from an urban school.	Garmin Vivofit	Effectiveness	Two-arm pilot study (two experimental groups).	Both groups received the Garmin Vivofit. IG1^b^: Daily goal to achieve. IG2 ^c^: Achieve as many steps as they could/wanted.	8 weeks	IG1: 4 IG2: 3	No difference in days spent in MVPA between IG1 and IG2. Adolescents in IG2 took more steps/day than adolescents in IG1.
Buchele Harris et al. [48]	Not specified.	116 adolescents (10–11 years) from two schools.	Fitbit Charge HR	Effectiveness	Three-arm quasi-experimental (2 intervention) and 1 control).	IG1: Received a Fitbit Charge HR. IG2: Received a Fitbit Charge HR, took part in a step challenge, and a series of 20 video-based PA exercises. CG^d^: No intervention.	20 days	IG1: 7 IG2: 11 CG:0	Participants in IG2 took 2197 more daily steps, spent more time being fairly and very active and less time being sedentary than participants in IG1.
Drehlich et al. [49]	Melbourne, Australia	124 inactive adolescents (13–14 years), from low SES schools.	Fitbit Flex	Acceptability, feasibility	Two-arm randomised controlled trial but assessed one-arm’s experience using the wearable (thus a one-arm feasibility study).	Received a Fitbit Flex and behaviour change resources via a private, research-moderated Facebook group.	12 weeks	N/A^e^	N/A
Evans et al. (study 1; [50])	Rhode Island, USA	32 children (M = 10 years) recruited from two fifth-grade classrooms in a low-income urban community.	Fitbit Zip	Effectiveness	One-arm pilot study.	Participants received a Fitbit Zip and a handout stating 10 ways to increase their step count.	4 weeks	6	Participants took a mean of 10,000 steps per day and increased their mean daily steps by 381 from week 1 to week 4.
Evans (study 2; [50])	Rhode Island, USA	42 adolescents (M = 12.3 years) recruited from four classrooms in a low-income urban community.	Fitbit Charge	Acceptability, feasibility, effectiveness	Three-arm open controlled pilot (school-level intervention (two groups) and control group).	IG1: Received a Fitbit Charge and 6 weekly 40 minute PA sessions led by their teachers and the research team. Incentives were provided to those who met their daily step goal. Participants took part in competitions between pupils and teachers. IG2: Provided with a Fitbit Charge. Did not receive incentives, competitions, or goal setting. CG: No intervention.	6 weeks	IG1: 10 IG2: 4 CG: 0	Mean daily step count and MVPA did not differ between IG1, IG2 and CG.
Galy et al. [51]	Lifou Island, New Caledonia	24 adolescents (12–14 years) from a rural school.	Misfit Shine 2	Effectiveness	One-arm pilot study.	Received a Misfit Shine 2 and a self-paced 8 module (1 h each) learning app (iEngage).	4 weeks	8	Participants averaged 64 mins/day, 14 mins/day and 7 mins/day of LPA, MPA and VPA, respectively. Participants achieving at least 11,000 steps/day increased from 48% of days (week 1) to 54% of days (week 4).
Gaudet et al. [52]	Canada	46 adolescents (13–14 years).	Fitbit Charge HR	Effectiveness	Cross-over pilot study (intervention and control period).	Received a Fitbit Charge HR.	7 weeks	IG: 6 CG: 0	No difference in MVPA between groups.
Götte et al. [53]	Not specified	40 adolescents (M = 14.7 years), with cancer.	Fitbit One or Flex	Effectiveness	Two-arm prospective, quasi-experimental study (intervention and control group).	IG: Received a Fitbit One (*n* = 5) or Flex (*n* = 35), encouraged to meet PA goals, and received an at-home exercise plan consisting of 5–7 exercises to improve strength, coordination and endurance. CG: Wait-list.	6–8 weeks	IG: 7 CG: 0	Participants increased their mean daily step count and active minutes by 1580 and 11.8, respectively. Steps, achievement of step goals, active minutes and achievement of active minute goals did not differ between groups.
Guthrie et al. [54]	Morgantown WV, Mountain View CA, and Vista CA, USA	182 adolescents (13–14 years) from three study sites.	Zamzee	Effectiveness	Three-arm pilot multi-site randomized controlled experiment (intervention group, active control and passive control group).	IG: Received the Zamzee and access to the website (PA progress and rewards). CG1 (Active control): Received Zamzee (no access to website) and an active game (Dance Dance Revolution). CG2 (Passive control): Received a Zamzee (no access to website).	6 weeks	IG: 9 CG1: 1 CG2: 1	Participants in the IG demonstrated an average of 15.26 minutes of MVPA/day, which was 67% and 49% greater than those in CG1 (9.12 mins) and CG2 (10.27 mins).
Hayes and van Camp [23]	USA	Six girls (8 years) from one school.	Fitbit Tracker (first model)	Effectiveness	Cross-over study (intervention and control period).	Received a Fitbit and step goal during seven recess sessions (20 min) and a final recess session where three step goals were set.	8 sessions (20 min each)	IG: 12 CG: 0	Participants took 47% more steps, and 21% more time in MVPA, during intervention than control periods.
Heale et al. [55]	Toronto, Canada	31 patients (12.8–18.6 years) with juvenile idiopathic arthritis. *n* = 28 in analysis.	Misfit Flash	Effectiveness	One-arm pilot study.	Received a Misfit Flash and set a daily PA goal.	4 weeks	4	Participants did not demonstrate a significant difference in mean METs/day or MVPA blocks/day from baseline to week 5.
Hooke et al. [25]	Midwest and South-eastern region, USA	16 children (6–15 years) with acute lymphoblastic leukemia.	Fitbit One	Effectiveness	One-arm pilot study.	Received a Fitbit One. A research nurse emailed participants and their parent(s) daily with their daily step count and PA levels, along with a brief message of encouragement.	17 days intervention	10	No significant changes in daily steps.
Kerner et al. [56]	North-west England	62 adolescents (14–15 years) from one school. *n* = 28 in analysis.	Fitbit Charge HR	Effectiveness	One-arm pilot study.	Received a Fitbit Charge HR, with instructions on how to use.	5 weeks	8	Participants decreased their daily MVPA by 9.53 minutes/day from pre- to post-intervention.
Kerner and Goodyear [57]	Southeast and northwest England	84 adolescents (13–14 years) from two schools.	Fitbit Charge	Acceptability, feasibility	One-arm feasibility study.	Received a Fitbit Charge.	8 weeks	N/A	N/A
Knox et al. [58]	Nottingham and Leicester, England.	49 participants (9–12 years) diagnosed with type 1 diabetes mellitus.	Polar Active	Acceptability, Feasibility, Effectiveness	Two-arm randomised controlled trial (intervention and control hospital site)	IG: Received a Polar Active, access to the Steps to Active Kids with Diabetes (STAK-D) website, and usual care for diabetes. CG: Received usual care for diabetes.	6 months	IG: 9 CG: 0	Mean change in daily steps from baseline to post-intervention and follow-up were 1162 and 899 steps/day greater in the IG than the CG. Changes in self-reported PA, MPA, VPA, and easy minutes did not differ between the IG and CG. PAQ sedentary scores significantly decreased in the IG from pre- to post-intervention (but not follow-up).
Larson et al. [59]	Mountain West region, USA	187 children (8–10 years) from two schools. *n* = 159 in analysis.	New Lifestyles NL−1000	Effectiveness	Two-way quasi-experimental (intervention school and control school)	IG: Received the NL−1000 and the “Fit ‘n’ Cool Kids” intervention. CG: Received no intervention.	16 school days.	IG: 11 CG: 0	Intervention participants took significantly more steps and spent more time in MVPA than the CG. Participants in the IG had a mean increase of 6.5 minutes of MVPA from pre- to post-intervention.
Mackintosh et al. [60]	Australia	25 families (36 children; 7–12 years).	Kidfit	Acceptability, feasibility	One-arm feasibility study.	Received a Kidfit.	4 weeks	N/A	N/A
Marttinen et al. [61]	Northeast, USA	13 adolescents (M = 12.15 years).	MOVband	Acceptability, feasibility	One-arm feasibility study.	Received a MOVband and took part in the F.I.T Unit, which delivered 12 fitness-based lessons while integrating academic subjects to develop a fitness plan and used PA data to develop fitness plans.	12 lessons	N/A	N/A
Masteller et al. [62]	Not Specified	16 children (M = 8.6 years).	Sqord, MOVband, and Zamzee.	Acceptability, feasibility	One-arm feasibility study.	Participants wore all three devices simultaneously and were instructed to spend ≥10 mins/day on each partnering website.	4 days	N/A	N/A
Mendoza et al. [63]	Seattle Children’s Hospital, USA	59 cancer survivors (14–18 years).	Fitbit Flex	Acceptability, feasibility, effectiveness	Two-arm, unblinded, RCT (hospital site-level intervention and control group).	IG: Received a Fitbit Flex (encouraged to reach daily step goal), voluntary participation in a researcher-moderated Facebook group, and usual care. A researcher sent text messages every other day to encourage and remind participants to reach their PA goal. CG: Received usual care.	10 weeks	IG: 12 CG: 1	Mean change in MVPA and sedentary time did not differ between the IG and CG.
Müller et al. [64]	Bavaria and Baden- Württemberg, Germany	59 children (M = 7.1 years).	Garmin Vivofit jr	Effectiveness	One-arm pilot study.	Received a Garmin Vivofit jr (partnering app was monitored by parents).	7 days	9	Participants took a mean daily step count of 12,202 and participated in 83 minutes of daily MVPA.
Nation-Grainger [65]	England	10 male adolescents (14–15 years) with low PA motivation from 1 school.	Samsung Galaxy Gear HR	Acceptability, feasibility, effectiveness	Two-arm quasi-experimental (school-level intervention and control group).	IG: Wore a masked Samsung Galaxy Gear HR in 6 PE lessons (1 per week). Received biofeedback after each PE lesson. CG: Wore a masked Samsung Galaxy Gear HR in 6 PE lessons (1 per week). Did not receive any biofeedback.	6 PE lessons (1 per week)	IG: 2 CG: 1	No difference in calories expended between IG and CG.
Phan et al. [66]	Two tertiary care weight management clinics (Mid-Atlantic and South Atlantic), USA	88 adolescents (13–17 years, BMI ≥85 th percentile).	Not reported	Effectiveness	Two-way randomised pilot study (two intervention groups).	Received standard weight management treatment and a wearable device and encouraged to increase their step goals. IG1: Only the adolescent received a wearable device. IG2: Adolescent and their parent received a wearable device,	3 months	IG1: 11 IG2: 11	Daily steps, MVPA, and calories expended did not differ between IG1 and IG2.
Remmert et al. [67]	California, USA	20 inactive adolescents (M = 12 years). *n* = 15 in analysis.	Fitbit Flex 2	Acceptability, feasibility, effectiveness	Two-way non-randomised pilot study (two intervention groups at school-level).	IG1: Received a Fitbit Flex 2 and acceptance-based behavioural counselling combined with preferred-intensity exercise for 30 minutes. IG2: Received a Fitbit Flex 2 only.	12 weeks	IG1: 6 IG2: 3	Participants in IG1 increased their daily steps and MVPA by 125 and 0.99 mins/day, respectively From pre- to post-intervention, IG1 increased their minutes of MVPA/day by 7.25 and IG2 increased their minutes of MVPA/day by 11.99.
Ridgers et al. [68]	Melbourne, Australia	60 adolescents (13–14 years) from three secondary schools.	Fitbit Flex	Acceptability, feasibility	One-arm feasibility study.	Received a Fitbit. No other information (e.g., goal setting, how often to wear the device) was provided.	6 weeks	N/A	N/A
Ruotsalainen et al. [69]	Northern Finland	46 overweight or obese adolescents (13–14 years).	Polar Active	Effectiveness	Three-arm randomised controlled trial (2 intervention groups and 1 control group).	IG1: Received a physiotherapist moderated Facebook-delivered lifestyle counselling, to discuss how to motivate participants to increase PA. IG2: Received the Facebook-delivered lifestyle counselling and a Polar Active. CG: No intervention.	12 weeks	IG1: 8 IG2: 11 CG: 0	Changes in LPA, MPA, MVPA and VPA, from baseline to post-intervention did not differ between IG1, IG2 and CG. Participants in IG2 (but not IG1) were less sedentary, than the CG, at post-intervention.
Schaefer et al. [32]	Yolo County, CA, USA	24 children (7–10-years).	Four devices, of which two were considered wearables (Polar Active and SenseWear ArmBand)	Acceptability, feasibility	One-arm feasibility study.	Wore the SenseWear Armband and Polar Active for 1 week each.	2 weeks	N/A	N/A
Schaefer et al. [33]	Northern California, USA	34 adolescents (11–12 years) recruited from a school with “high poverty”. *n* = 24 in analysis.	Fitbit One	Acceptability, feasibility	One-arm feasibility study.	Received the Fitbit One during an afterschool program, then all day, every day for 5 months.	6 months	N/A	N/A
Schoenfelder et al. [70]	Washington, USA	11 adolescents (14–18 years), with ADHD.	Fitbit Flex	Effectiveness	One-arm pilot study.	Received a Fitbit Flex, with a daily step goal, and joined a private Facebook group, where they were encouraged to post in the group (e.g., encourage participants and post their Fitbit data).	4 weeks	11	Participants increased their daily step count by 107 steps/day.
Sharaievska et al. [71]	Appalachia, USA	11 families from a rural community, with one to three children (7–13 years) per family.	Fitbit Zip	Acceptability, feasibility	One-arm feasibility study.	Family members received a Fitbit Zip.	2 weeks	N/A	N/A
Slootmaker et al. [24]	Amsterdam, Netherlands	87 inactive adolescents (13–17 years) from five schools. *n* = 68 at follow-up.	PAM	Effectiveness	Two-arm randomised controlled trial (intervention and control group).	IG: Received the PAM and its partnering website (PAM COACH). CG: Received a single information brochure with general PA recommendations.	3 months	IG: 8 CG: 0	No difference in pre- and post-intervention (3 month) and follow-up (8 month) LPA, VPA and MVPA between the IG and CG. Compared to the CG, boys in the IG reduced their sedentary time by 1801 minutes/week from pre-intervention to 8-month follow-up, and girls in the IG increased their weekly MPA by 411 minutes/week from pre-intervention to post-intervention (but not follow-up).
Yoost et al. [72]	USA	34 adolescents aged 13–18 years (BMI >95th percentile). *n* = 24 in analysis.	Fitbit Charge	Acceptability, feasibility, effectiveness	One-arm pilot study.	Received standardised diet and exercise counselling, and a Fitbit Charge.	6 months	7	Participants took a mean of 5101 steps per day throughout the intervention. Participants decreased their average daily step from 6462 steps/day (month 1) to 5101 steps/day (month 3).

^a^ body mass index, ^b^ intervention group 1, ^c^ intervention group 2, ^d^ control group, ^e^ not applicable.

**Table 3:** Risk of bias results for individual effectiveness studies.

Citation	Random Allocation	Minimal Missing Data	Analysed According to Group	Representative Sampling	Timing of Outcome Assessments	Validity of Wearable	Reliability of Wearable	Independent of Wearable Manufacturer	Summary Score	Rob Level
Bianchi-Hayes et al. [46]	0	1	0	0	0	1	1	1	4	Medium
Bronikowski et al. [47]	1	1	1	1	1	0	0	1	6	Low
Buchele Harris et al. [48]	0	1	1	1	1	0	0	1	5	Medium
Evans et al. (study 1; [50])	0	1	1	0	1	0	0	1	4	Medium
Evans et al. (study 2; [50])	0	1	1	0	1	0	0	1	4	Medium
Galy et al. [51]	0	1	1	0	1	0	0	1	4	Medium
Gaudet et al. [52]	1	1	1	1	1	0	0	1	6	Low
Götte et al. [53]	0	1	1	0	1	0	0	1	4	Medium
Guthrie et al. [54]	1	1	1	1	1	1	0	1	7	Low
Hayes & van Camp [23]	0	0	0	0	0	0	1	1	2	High
Heale et al. [55]	0	0	0	0	0	0	0	1	1	High
Hooke et al. [25]	0	0	0	0	0	0	0	1	1	High
Kerner et al. [56]	0	0	0	0	0	0	0	1	1	High
Knox et al. [58]	1	0	1	1	1	0	0	1	5	Medium
Larson et al. [59]	1	0	1	0	1	1	0	1	5	Medium
Mendoza et al. [63]	1	1	1	0	1	0	0	1	5	Medium
Müller et al. [64]	0	1	0	1	0	1	0	1	4	Medium
Nation-Grainger [65]	1	1	1	0	1	1	0	1	6	Low
Phan et al. [66]	1	1	1	0	1	1	0	1	6	Low
Remmert et al. [67]	0	0	1	1	1	0	0	1	4	Medium
Ruotsalainen et al. [69]	1	1	1	1	1	0	0	0	5	Medium
Schoenfelder et al. [70]	0	1	0	1	0	0	0	1	3	Medium
Slootmaker et al. [24]	1	1	1	1	1	0	0	1	6	Low
Yoost et al. [72]	0	0	0	0	0	0	0	1	1	High

**Table 4:** Themes, findings, and supporting quotations identified in the thematic synthesis.

Review Aim	Analytical Theme	Descriptive Theme	Subthemes	Number of Supporting Studies	Findings and Supporting Quotations
Feasibility	Perceived facilitators and barriers of using a wearable may impact device use	Factors impacting the use of wearables	Device technical difficulties	8	Some adolescents reported general barriers to technology, such as access to a computer [61] or the Internet [33], that may limit device use. Some participants reported difficulties with charging and syncing devices (*n* = 3): “They didn’t charge properly” (Kidfit [60]). Other participants reported daily syncing and charging as burdensome: “Sometimes I forgot, it’s like getting a little bit annoying to have to like do it every day” (Fitbit Flex [49]).
			Device design impacts wearability	10	Some wrist-worn devices were deemed uncomfortable and bulky: “The wristband wasn’t comfortable” (Kidfit [60]). “I kind of got annoyed with it at the end because of the bulkiness… It was a factor in like I didn’t really want to have to wear it” (MOVband [61]). However, participants were worried they would lose the Fitbit One due to its small size: “I didn’t like it because it was too tiny, I thought I was going to lose it” [33].
			Removal for sports and daily activities	7	Some participants disliked that the wearable was unable to capture their activity during sports: “Because I kind of like it when you can see that you’re getting really high [step counts] and not being able to wear it during sporting events. I wasn’t able to see what I was really getting and how high I could really get” (MOVband [61]). Participants reported forgetting to wear the device following removal due to daily activities, such as showering and getting changed: “Now that it’s not waterproof, I like forget to put it back on after a shower” (Polar Active [58]). “Changed into my pyjamas I was like, ‘Oh, I forgot I had that’” (Fitbit One [33]).
Acceptability	Affective attitude: Feelings towards using wearables	Participants’ feelings of enjoyment, boredom, frustration, and distrust towards using wearables.	Enjoyment of using wearables	8	Participants enjoyed using the devices, which may be attributed to their gamification components: “It was fun to compare steps” (Fitbit One [33]). “It felt like a computer game” (Samsung Galaxy Gear HR [65]) “It was fun. It’s almost like a game” (MOVband [61]).
			Novelty effect	6	Participants reported a potential novelty effect of using the device, which may be attributed to boredom: “It’s fun for a bit … the novelty rubs off and just... oh well, I don’t really care anymore” (Fitbit Flex [49]). “I used it for the first 4 weeks, then just gave up” (Fitbit Charge [57]). One parent reported their child was not interested in owning a device after the study: “I said, ‘oh would you want to wear them, like would you want one of your own?’ They said ‘no, we’ve kind of used it now’” (Kidfit [60]).
			Questions regarding the integrity of wearables	4	Some participants admitted to testing the integrity of the device by shaking the device or counting their steps: “I tried to count actually how many steps I do, and then I looked at the Fitbit, and it was like 16 off” (Fitbit One [33]). “I would like shake it to see if it’s working and sometimes it wouldn’t” (Fitbit Flex [49]). One adolescent suggested people could “cheat” their PA data, by shaking the device: “But sometimes people can cheat on that, I think” (Fitbit Flex [49]).
			Disappointment due to child restrictions and parental control	2	Child restrictions resulted in feelings of disappointment in younger participants: “My mom feels like she didn’t really want this [software] on my laptop” (MOVband [61]). “Bit disappointed that she couldn’t access her own information … because they’d want to see what they’d scored” (Kidfit [60]).
	Perceived effectiveness and intervention coherence: wearables perceived ease of use, interpretation of PA outputs and impact on PA	Wearables ease of use, understanding of PA outputs, and perceived impact on PA varies between devices and individuals.	Understanding how to use wearables and interpret PA outputs	6	Participants reported wearables were easy to use, and PA outputs were easy to understand: “It was kind of easy to understand. It wasn’t confusing at all” (Sqord [62]). However, some participants reported a lack of understanding of how to use device features: “I was trying to like add more onto goals and stuff I found it like hard to use and I just like stopped using it” (Fitbit Flex [49]). “I sometimes look at the challenges but I don’t really know what they are”, “It’s really, really confusing how to get sqoins and stuff” (Sqord [62]).
			A perceived increase in PA levels	8	Some adolescents suggested the mere presence of the device made them more active: “Just knowing it’s on your wrist, it makes me want to be more active” (Fitbit Flex [68]). Most participants referred to a change in lifestyle: “So, we knew [due to the feedback report] we needed to go out a bit more on a Sunday, which we do actually do quite a bit now” (Polar Active [58]). “I would like try to do extra, like offer to take the dog out instead of complaining about it” (Fitbit One [33]). However, one adolescent believed this increase in PA would diminish when they no longer had access to the device: “Cuz I won’t see the results that I done” (Samsung Galaxy Gear HR [65]).
			Wearables do not impact PA levels	3	Some participants reported the wearable did not impact their PA levels. However, these participants perceived themselves to be active, and used the device to confirm their beliefs about their active lifestyle: “We were already pretty active so I don’t know” (Fitbit Zip [71]). “I think it just showed me what I was doing” (MOVband [61]).
Mechanisms of action	Perceived mechanisms of action underlying wearables impact on PA	Wearables may motivate or discourage PA via BCTs: feedback, self-monitoring and goal setting, competition, and rewards and incentives.	Feedback, self-monitoring, and goal-setting	9	Participants used immediate feedback to increase their PA levels, and awareness of their PA levels: “When it says get off the couch, he did respond to that” (Kidfit [60]). “Yeah, it makes you so aware of how many or how active you are and then you wanna try to harder” (Fitbit Flex [49]). Participants disliked feedback displayed as a visual representation, or that was only displayed via the partnering app: “What was the flower about?” (Fitbit One [33]) “Didn’t like the fact, unlike this [Fitbit] where you can see your steps instantaneously” (Kidfit [60]). Feedback promoted the use of self-monitoring and goal setting to increase PA. Participants reported a conscious effort to increase their PA to reach their PA goal, or when achieved, increase their PA goal: “In two days, I did 15 miles. My goal was, the next day, to do 6 more miles”, “I want to reach my goal” (MOVband [61]). However, some adolescents reported that predetermined goals (10,000 steps/day) may create feelings of pressure and guilt: “You can feel under pressure to do a certain amount of steps or to be better than what you’re maybe capable of” (Fitbit Charge [57]).
			Competition with the self and others	6	Participants suggested that “beating” their previous score, or their friends’ score, encouraged them to be more physically active: “I wanted to beat my score”, “I always tried to push myself to the next lesson … to try and get a higher mark” (Samsung Galaxy Gear HR [65]). Parents suggested that children may enjoy collective school competitions: “It would be great to have a competition between the classes, rather than amongst each individual kid, because then they’re helping each other along” (Kidfit [60]). However, 3 studies reported that competition may create feelings of pressure and guilt: “Some people maybe feel peer pressure to do fitness, to keep their steps and stuff up”, “You can sometimes feel guilty … I couldn’t let someone else beat me” (Fitbit Charge [57]).
			Rewards and incentives	3	Participants reported enjoying rewards and incentives, including social rewards: “I really like that, um, you get rewards”, “I like how you can “like” things because then it makes me feel good when people like my stuff because it makes me feel happy” (Zamzee [62]). However, some participants suggested virtual rewards, such as badges, were not considered an incentive to be physically active: “It’s not like a huge achievement or anything” (Fitbit Flex [68]).

## Data Availability

Not applicable.

## Supplementary Materials

The following are available online at: https://www.mdpi.com/article/10.3390/ijerph18126211/s1, Table S1: Search strings and results, Table S2: Risk of bias criteria, descriptions, and examples; Table S3: Physical activity measures, and classifications, used in effectiveness studies.
