# Supplementary material for: The Acceptability, Feasibility, and Effectiveness of Wearable Activity Trackers for Increasing Physical Activity in Children and Adolescents: A Systematic Review
Source: Int J Environ Res Public Health. 2021 Jun 8;18(12):6211. doi: 10.3390/ijerph18126211 (PMC8228417; doi:10.3390/ijerph18126211)
Supplement: Supplementary file 1 [file ijerph-18-06211-s001.zip › ijerph-1201785-supplementary.pdf]

SUPPLEMENTARY MATERIALS

Table S1. Search strings and results

Database	Search String	Number of studies identified*
PubMed	((((((electronic track* OR electronic activ* AND track* OR electronic activ* AND monitor* OR electronic fitness track* OR wearable device OR wearable act* AND track* OR consumer wearable OR wearable OR wearable tech* OR fitness track* OR activity monitor OR activity track*))) AND ((Intervention OR trial OR feasibility OR pilot OR acceptability OR program))) AND ((Child* OR Adolescen* OR youth OR teen* OR young person OR young people OR school child* OR family OR families OR parent* OR caregiver OR mother OR father OR home-based OR parent-child OR parent-adolescent))) AND ((Physical act* OR Energy expenditure OR MVPA OR steps)))	1234
Web of Science	TS=(electronic track* OR electronic activ* AND track* OR electronic activ* AND monitor* OR electronic fitness track* OR wearable device OR wearable act* AND track* OR consumer wearable OR wearable OR wearable tech* OR fitness track* OR activity monitor OR activity track*) AND TS=(Intervention OR trial OR feasibility OR pilot OR acceptability OR program) AND TS=(Child* OR Adolescen* OR youth OR teen* OR young person OR young people OR school child* OR family OR families OR parent* OR caregiver OR mother OR father OR home-based OR parent-child OR parent-adolescent) AND TS=(Physical act* OR Energy expenditure OR MVPA OR steps)	2740
Scopus	(electronic track* OR electronic activ* AND track* OR electronic activ* AND monitor* OR electronic fitness track* OR wearable device OR wearable act* AND track* OR consumer wearable OR wearable OR wearable tech* OR fitness track* OR activity monitor OR activity track*) AND (Intervention OR trial OR feasibility OR pilot OR acceptability OR program) AND (Child* OR Adolescen* OR youth OR teen* OR young person OR young people OR school child* OR family OR families OR parent* OR caregiver OR mother OR father OR home-based OR parent-child OR parent-adolescent) AND (Physical act* OR Energy expenditure OR MVPA OR steps)	140
SPORTDiscus	AB ((electronic track* OR electronic activ* AND track* OR electronic activ* AND monitor* OR electronic fitness track* OR wearable device OR wearable act* AND track* OR consumer wearable OR wearable OR wearable tech* OR fitness track* OR activity monitor OR activity track*)) AND AB ((Intervention OR trial OR feasibility OR pilot OR acceptability OR program)) AND AB ((Child* OR Adolescen* OR youth OR teen* OR young person OR young people OR school child* OR family OR families OR parent* OR caregiver OR mother OR father OR home-based OR parent-child OR parent-adolescent)) AND AB ((Physical act* OR Energy expenditure OR MVPA OR steps))	74

PsycINFO	(electronic track* OR electronic activ* AND track* OR electronic activ* AND monitor* OR electronic fitness track* OR wearable device OR wearable act* AND track* OR consumer wearable OR wearable OR wearable tech* OR fitness track* OR activity monitor OR activity track*) AND (Intervention OR trial OR feasibility OR pilot OR acceptability OR program) AND (Child* OR Adolescen* OR youth OR teen* OR young person OR young people OR school child* OR family OR families OR parent* OR caregiver OR mother OR father OR home-based OR parent-child OR parent-adolescent) AND (Physical act* OR Energy expenditure OR MVPA OR steps)	127
ProQuest	all(electronic track* OR electronic activ* AND track* OR electronic activ* AND monitor* OR electronic fitness track* OR wearable device OR wearable act* AND track* OR consumer wearable OR wearable OR wearable tech* OR fitness track* OR activity monitor OR activity track*) AND all(Intervention OR trial OR feasibility OR pilot OR acceptability OR program) AND all(Child* OR Adolescen* OR youth OR teen* OR young person OR young people OR school child* OR family OR families OR parent* OR caregiver OR mother OR father OR home-based OR parent-child OR parent-adolescent) AND all(Physical act* OR Energy expenditure OR MVPA OR steps)	438

* Before removing duplicates

Table S2. Risk of bias criteria, descriptions, and examples

Criterion	Description	Example
Participants were randomly allocated.	"Yes" if the author(s) states, or provides evidence, that participants were randomly allocated.<br><br>"No" if there was no mention of random allocation, if the author(s) stated participants were not randomly allocated, or the study had one intervention arm.	Yes: "Group were selected randomly"<br><br>No: "Participants were assigned…"
Minimal missing data (an adequate proportion of participants had complete data for the outcome variable).	"Yes" if ≥80% of the original sample had complete data for PA measures at each time point.<br><br>"No" if more than 20% of the original sample had missing data for PA measures at least one time point.	Yes: "Out of the 87 participants completing baseline measures, 79 provided completed follow-up data".<br><br>No: "70% of participants provided valid accelerometry data".
Data were analysed according to group allocation.	"Yes" if the author(s) stated the results were analysed according to group allocation and the results were presented according to group allocation.<br><br>"No" if data were pooled together and not analysed according to group allocation, or data were analysed according to subgroups (e.g., sex), rather than intervention group allocation.<br>"No" if the study had one intervention arm.	Yes: "The intervention group showed an average increase in MVPA of 15 minutes per day compared with the control group, which increased their MVPA per day by an average of 1 minute".<br><br>No: "All participants increased their steps by an average of 2000 per day, from pre- to post-intervention", but the author(s) do not report individual results for the intervention and control group.
The study population was representative of the population of interest.	"Yes" if there were clear justifications for the study population in the introduction and the author(s) stated their attempt to create a representative sample of the population in their methods (e.g., total number approached, how the sample reflected the population of interest, how sample characteristics were measured).	Yes: "PA levels were monitored for 2 weeks by means of a PA monitor and a PA questionnaire. Based on these 2 weeks, the study population (*n* = 286) was divided in an "active" (most active 50% of the population) and "inactive" (least active 50%) group".

	"No" if there was no clear justification for the study population in the introduction or methods section.<br><br>"No" if there was no information provided regarding how the author(s) attempted to recruit a representative sample of the population of interest (e.g., number approached).	No: "Boys with low participation levels in P.E.", when the author(s) provide no justification for just using boys or how they measured low participation levels in P.E.<br><br>No: "Eligible participants were receiving a cycle of maintenance chemotherapy", but the author(s) provide no information regarding how the sample was recruited to ensure it was representative.
The timing of outcome assessments were similar in all groups.	"Yes" if the author(s) reported outcome assessments took place within the same time scale in all groups.<br><br>"No" if the author(s) reported outcome assessments did not take place within the same time scale in all groups.<br><br>"No" if the study had one intervention arm.	Yes: "Post-intervention PA measures were taken within 1 week of the intervention end date, for intervention and control participants".<br><br>No: "Post-intervention PA measures were taken 1 week after the intervention end date for the intervention group, but 2 weeks after for the control group due to time constraints".
The study reported the validity of the wearable used.	"Yes" if the study provided an appropriate reference to an original study, confirming the validity of the device in participants representative of the study population (e.g., same age group, weight status) or provided data confirming the validity of the device in the study population.<br><br>"No" if the study did not provide an appropriate reference to an original study or provide data confirming the validity of the device.<br>"No" if the study provided a reference to an original study confirming the validity of the device, but in a sample that was not representative of the study population (e.g., adults, a non-clinical sample in a clinical study).	Yes: "Previous research has reported this device to be a valid measure of PA, compared to an accelerometer, in 6 to 7-year-olds *(*original reference*)"*.<br><br>No: "Previous research has reported this device to be a valid measure of PA, compared to an accelerometer, in elderly populations *(*original reference*)"*.
The study reported the reliability of the wearable used.	"Yes" if the study provided an appropriate reference to an original study, confirming the reliability of the device in participants representative of the study population (e.g., same age	Yes: "Previous research has reported this device to be a reliable measure of PA, compared to an accelerometer, in 6 to 7-year-olds *(*original reference*)"*.

	group, weight status) or provided data confirming the reliability of the device in the study population.<br><br>"No" if the study did not provide an appropriate reference to an original study or provide data confirming the reliability of the device.<br><br>"No" if the study provided a reference to an original study confirming the reliability of the device, but in a sample that was not representative of the study population (e.g., adults, a non-clinical sample in a clinical study).	No: "Previous research has reported this device to be a reliable measure of PA, compared to an accelerometer, in elderly populations *(*original reference*)"*.
The study was conducted independently of the wearable manufacturer.	"Yes" if the author(s) did not report any conflicts of interest it was assumed the study was conducted independent of the wearable manufacturer.<br><br>"Yes" if the author(s) explicitly reported the study was conducted independently of the wearable manufacturer.<br><br>"No" if the author(s) reported the study was conducted in partnership with the wearable manufacturer (e.g., employee, partnership, promotion) or were loaned the devices for the study.	Yes: "The authors declare no conflicts of interest".<br><br>No: "The devices used within this study were gifted by **device brand** for promotional purposes".<br><br>No: "**device brand** loaned some activity monitors for the examinations".

Table S3. Physical activity measures, and classifications, used in effectiveness studies

Citation	PA measurement tool(s)	Outcome(s) reported	Measurement time point(s)	Measurement classifications
Bianchi-Hayes et al.[46]	Jawbone UP MOVE.	Achievement of step and active minute goals and daily active minutes.	Throughout the intervention.	**Achievement of step and active minute goals**: the days when participants reached their personalised goal or those set for their age (12,000 steps and 60 active minutes for adolescents).<br>**Active minutes:** not specified
Bronikowski et al.[47]	Physical Activity Screening Measure.<br>**Q1.** Over the past 7 days, on how many days were you physically active for a total of at least 60 min per day?<br>**Q2.** Over a typical or usual week, on how many days are you physically active for a total of at least 60 min per day?	Daily steps and MVPA.	Steps (8-week post-intervention).<br><br>MVPA (baseline and 8-week post-intervention).	**MVPA:** (Answer to Q1 + answer to Q2)/2.
Buchele Harris et al.[48]	Fitbit Charge HR.	Steps during school hours, sedentary time, LPA, fairly active, and very active minutes.	Throughout the intervention, during school hours (Mon–Fri).	**Sedentary time:** 1 MET[a]<br>**LPA**: 2–3 METs<br>**Fairly active minutes**: 4–6 METs<br>**Very active minutes:** >6 METs.
Evans et al. study 1[50]	Fitbit Zip.	Daily steps.	Throughout the intervention.	
Evans et al. study 2[50]	Fitbit Charge and SenseWear Armband Mini.	Daily steps (Fitbit Charge) and MVPA (SenseWear Armband Mini).	Steps (throughout the intervention, baseline and 6 weeks post-intervention)<br><br>MVPA (baseline and 6 weeks post-intervention).	**MVPA:** >4 METs

Galy et al.[51]	Misfit Shine 2.	Daily steps, achievement of step goals, LPA, MPA, and VPA.	Steps, LPA, MPA and VPA.	**LPA:** <2 calories<br>**MPA:** 2–3.5 calories.<br>**VPA:** >3.5 calories
Gaudet et al.[52]	Fitbit Charge HR and Actical accelerometer (hip worn).	MVPA.	Baseline, throughout, 7 weeks and 14 weeks.	**MVPA:** ≥1500 cpm[b]
Götte et al.[53]	Fitbit One or Flex.	Daily steps, active minutes and achievement of step and active minute goals	Throughout the intervention: Time 1: during the intervention.<br><br>Time 2: during a 2-week intervention 3 months after the acute cancer treatment	**Active minutes:** not specified<br>**Achievement of step and active minute goals:** a deviation less than 30% from the defined goal
Guthrie et al.[54]	Zamzee.	MVPA.	Throughout the intervention.	**MVPA:** ≥4 METs (sum of 10 s of VMa values/day).
Hayes and van Camp[23]	Fitbit Tracker (first model; masked).	Steps during recess and MVPA.	Throughout intervention and control recess periods.	**MVPA:** >99 steps/min.
Heale et al.[55]	3day PA recall (recall activities and intensity of the activities performed in the past 3 days; segmented into 34 30 min time blocks (7:00 a.m. to midnight).	METs and MVPA.	Baseline, week 1 and 5-week follow-up.	**METs/day:** MET value assigned to each 30-minute block based on activity and levels of intensity reported)<br>**MVPA:** Number of blocks where energy expenditure was estimated at ≥3 METs
Hooke et al.[25]	Fitbit One (unmasked).	Daily steps.	Throughout the intervention.	
Kerner et al.[56]	ActiGraph GT9X triaxial accelerometer (non-dominant wrist).	MVPA.	Baseline and 5-week post-intervention.	**MVPA:** ≥2296 cpm

Knox et al.[58]	Polar Active and Physical Activity Questionnaire (PAQ).	Daily steps, active minutes, MPA and	Baseline, 8-week (during the	Not specified
Larson et al.[59]	New Lifestyles NL-1000 (unmasked).	Steps during school hours and MVPA.	Throughout the intervention (school hours only), baseline and 10-week follow-up (daily).	**MVPA:** >3 METs.
Mendoza et al.[63]	ActiGraph GT3X+ (hip worn).	Sedentary behaviour and MVPA.	Baseline and weeks 8–10 of the intervention.	Evenson PA intensity cut points
Müller et al.[64]	Garmin Vivofit jr.	Daily steps and MVPA.	Throughout the intervention.	**MVPA:** not specified.
Nation-Grainger[65]	Samsung Galaxy Gear HR (masked).	Calories expended during P.E lessons	Baseline and 6-week post-intervention.	
Phan et al.[66]	Wearable device utilised.	Daily steps, MVPA and daily calories expended.	Throughout the intervention.	**MVPA:** not specified.
Remmert et al.[67]	Fitbit Flex 2 and ActiGraph GT3X+ (non-dominant hip).	Daily steps and MVPA.	Steps (throughout the intervention).<br><br>MVPA (throughout the intervention, baseline and post-intervention: 10-weeks).	**MVPA:** Freedson PA intensity cut points
Ruotsalainen et al.[69]	Polar Active (objective measure) and questions from the "WHO Health Behaviours in School-Aged Children study": "Over the past 7 days, on how many days were you physically active for a total of at least 60 minutes per day?" (self-report measure).	Sedentary/ very LPA, LPA, MPA, VPA, and MVPA.	Baseline and post-intervention (12-weeks).	**Sedentary/very LPA:** <2 METs<br>**LPA:** 2-3.49 METs<br>**MPA:** 3.5–4.99 METs<br>**VPA:** 5–8 METs<br>**MVPA:** ≥3.5 METs and self-report (no. of days achieving 60 mins of daily MVPA)

Schoenfelder et al.[70]	Fitbit Flex.	Daily steps.	Throughout the intervention.	
Slootmaker et al.[24]	The Activity Questionnaire for Adolescents and Adults (AQuAA).	Sedentary time, LPA, MPA, VPA and MVPA.	Baseline, 3-month post-intervention and 8-month follow-up.	**Sedentary:** <2 METs<br>**LPA:** 2–5 METs<br>**MPA:** 5–8 METs<br>**VPA**: >8 METs<br>**MVPA:** ≥5 METs
Yoost et al.[72]	Fitbit Charge.	Daily steps and achievement of step goals.	Throughout the intervention.	**Achievement of step goals:** number of days achieving 10,000 steps/day.

[a] metabolic equivalent of task, [b] counts per minute
